# T Cell Exhaustion as a Regulated Differentiation Programme

**DOI:** 10.1002/eji.70205

**Published:** 2026-06-17

**Authors:** Marc Veldhoen, Cristina Ferreira

**Affiliations:** ^1^ Gulbenkian Institute for Molecular Medicine Lisbon Portugal; ^2^ Faculdade de Medicina da Universidade de Lisboa Lisbon Portugal

## Abstract

T cell exhaustion is now recognised as a structured, antigen‐driven differentiation programme rather than a state of cellular fatigue. Under sustained antigen exposure, CD8^+^ T cells progress through distinct, hierarchically organised differentiation states, initiated by progenitor exhausted cells (T_PEX_), which retain self‐renewal, multipotency, and responsiveness to immune checkpoint blockade. Continued stimulation drives differentiation into intermediate (T_EXint_) and terminally exhausted (T_EX term_) states, with T_EXint_ retaining greater effector capacity than T_EXterm_ despite both exhibiting restraint relative to functional effector T cells, alongside increasingly consolidated epigenetic architecture. Rather than reflecting immunological failure, exhaustion preserves long‐term antigen surveillance while limiting tissue damage. Convergence between exhaustion‐associated transcriptional modules and tissue‐resident memory (T_RM_) programmes highlights shared mechanisms of adaptation to restrictive microenvironments. Yet T_RM_ and T_EX_ arise in distinct contexts and are not interchangeable. Recognising exhaustion as a context‐dependent differentiation process reframes therapeutic strategies, in line with current evidence indicating that immune checkpoint blockade primarily acts by expanding and redirecting the T_PEX_ pool rather than reversing terminal exhaustion. This framework integrates insights from chronic infection, tumour immunology, and tissue adaptation.

## Graphical abstract

This review highlights T cell exhaustion as an adaptive differentiation trajectory, not dysfunction, showing how precursor, intermediate, and terminal exhausted states maintain antigen recognition while limiting tissue damage in chronic immune activation.

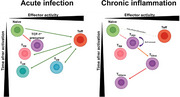

## 1 Introduction

Early descriptions of T cell exhaustion emerged from studies of chronic viral infection, where antigen‐specific CD8^+^ T cells progressively lost the ability to produce cytokines and displayed sustained expression of inhibitory receptors such as PD‐1, TIM‐3, and LAG‐3 [1, 2, 3, 4]. These observations led to the interpretation that exhausted T cells represented a dysfunctional or deteriorating population incapable of sustaining immune responses [5]. However, advances in single‐cell transcriptomics, lineage‐tracing, and chromatin profiling have revised this view [6, 7]. Exhaustion is now understood as a regulated differentiation programme that arises under persistent antigen stimulation and enables continued antigen surveillance while restraining excessive immunopathology.

To avoid confusion with other hyporesponsive states, we use exhaustion strictly to denote the antigen‐driven differentiation trajectory distinct from anergy (signal 1 in the absence of co‐stimulation) and senescence (replicative arrest with altered secretory profiles).

Exhaustion unfolds through a hierarchical series of differentiation states [8]. A progenitor exhausted population, T_PEX_, marked by TCF‐1 and intermediate PD‐1, retains self‐renewal, multipotency, and responsiveness to cytokine and co‐stimulatory signals. With continued antigen stimulation, T_PEX_ cells differentiate into intermediate exhausted cells (T_EXint_) with the greatest preserved effector potential, and subsequently into terminally exhausted cells (T_EX term_) characterised by limited proliferation, entrenched epigenetic landscapes, and strong inhibitory receptor expression. Distinguishing these states is essential for understanding functional heterogeneity, tissue localisation, and therapeutic responsiveness. Throughout the manuscript, we adopt a consistent state nomenclature that separates TCF‐1^+^ T_PEX_ from T_EXint_ and T_EXterm_ subsets, in line with emerging field guidance [9] (Table 1).

**TABLE 1:** Functional and molecular characteristics of activated CD8^+^ T cell subsets.

Subset	Surface markers	Key transcription factors	Metabolic characteristics	Proliferation	Effector function	Self‐renewal
Effector (T_eff_)	CCR5, CXCR3, CX3CR1	T‐bet, EOMES, RUNX3, BLIMP‐1	High glycolysis and glutaminolysis; low OXPHOS	High during expansion	High cytotoxicity and cytokine production	None
Central Memory (T_CM_)	CD62L, CCR7	TCF‐1, LEF1, BCL‐6, TLE3	FAO and OXPHOS dominant	Moderate	Moderate (rapid recall)	High
Effector Memory (T_EM_)	CXCR3	T‐bet, BLIMP‐1, RUNX3, EOMES^int^	Mixed glycolysis, FAO, and OXPHOS	Moderate	Moderate‐high	Limited
Tissue‐resident memory (T_RM_)	CD69, CD103, CD49a, CXCR6; often PD‐1/TIM‐3/TIGIT	Hobit, BLIMP‐1, AhR, RUNX3, Bhlhe40, BATF	Tissue‐adapted glycolysis–FAO balance	Low (homeostatic)	Low baseline; rapid local response upon re‐challenge	Local maintenance
Progenitor exhausted (T_PEX_)	TCF‐1^+^, PD‐1^int^, CXCR5^+^	TCF‐1, BCL‐6, LEF1	Balanced glycolysis, FAO, and OXPHOS	High proliferative potential	Low‐moderate	High
Intermediate exhausted (T_EXint_)	PD‐1, TIM‐3^lo/int^, TIGIT, CTLA‐4	TOX, BATF, IRF4, T‐bet^lo^, TCF‐1^lo^	Glycolysis increased during proliferation	Moderate	Moderate‐high	Limited
Terminal exhausted (T_EX‐term_)	PD‐1^hi^, TIM‐3^hi^, TIGIT, CTLA‐4	TOX, NR4A, EOMES^hi^, NFIL3	Reduced mitochondrial function; metabolic inflexibility	Low	Low	None

*Note*: Markers annotated as hi/int/lo indicate relative expression; ‘effector function’ refers to cytokine production and cytotoxic capacity as defined in the text; proliferation is steady‑state unless noted.

A key insight from recent lineage‐tracing studies is that TCF‐1^+^PD‐1^+^ precursor‐like cells arise very early after priming, even during acute infection. These cells represent a physiological intermediate capable of generating effector and memory populations when antigen is cleared, but they also provide the progenitor pool that sustains the exhaustion lineage when antigen persists [10, 11]. Thus, this precursor stage represents a flexible differentiation checkpoint whose outcome depends on antigen persistence, inflammatory context, and tissue environment rather than constituting an intrinsically exhausted state. Although exhaustion is distinct from other memory and effector fates, exhausted T cells share regulatory and metabolic features with tissue‐resident memory T (T_RM_) cells (Table 1). Both programmes involve transcriptional restraint, adaptation to cytokine‐restricted and nutrient‐limited environments, and upregulation of inhibitory receptors, yet these similarities reflect adaptive convergence to local tissue constraints rather than lineage equivalence.

**FIGURE 1:**
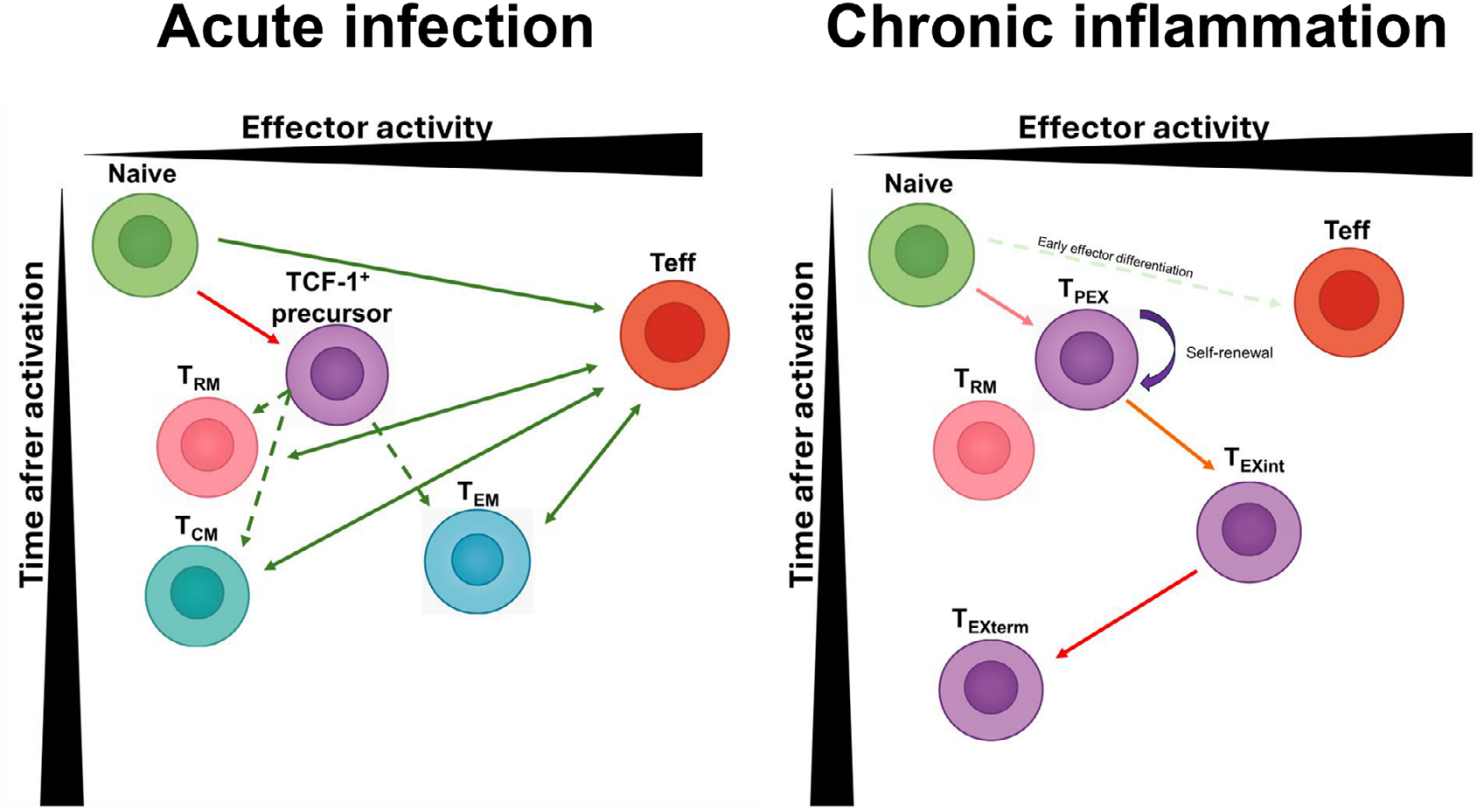
Differentiation trajectories of CD8^+^ T cells during acute and chronic antigen exposure. During acute infection or vaccination (left), naïve CD8^+^ T cells differentiate into effector (T_eff_) cells and diverse memory subsets, including central memory (T_CM_), effector memory (T_EM_), and tissue‑resident memory (T_RM_) cells. A small population of precursor‑like TCF‑1^+^PD‑1^+^ cells (T_PEX_‑like) can arise transiently after priming; these cells are not exhausted and can contribute to effector and memory formation once the antigen is cleared. Under chronic antigen exposure (right), persistent stimulation drives a hierarchical exhaustion trajectory in which self‑renewing T_PEX_ cells generate intermediate exhausted cells (T_EXint_) with partial effector capacity, followed by terminally exhausted cells (T_EXterm_) characterised by reduced proliferation, diminished cytokine production, entrenched epigenetic landscapes, and preserved antigen recognition. In this setting, differentiation into long‑lived memory subsets may be curtailed. Green arrows indicate productive differentiation pathways; red arrows indicate exhaustion‑associated trajectories under sustained antigen stimulation; dashed arrows indicate potential transitions.

Exhausted and resident cells arise under different antigenic and environmental pressures, and their trajectories diverge in ways that have important implications for therapeutic interventions. A central conceptual reframing is that exhaustion does not represent T cell “fatigue” or incompetence. Exhausted T cells remain sensitive to their cognate antigen and can exert sustained, calibrated effector functions in settings such as chronic infections or cancer, where continuous cytotoxic activity from effector cells would cause excessive tissue damage or lead to collapse of the response. Understanding exhaustion as an adaptive process supports focusing on preserving, expanding, or redirecting the T_PEX_ cell compartment rather than attempting to reprogramme T_EXterm_ cells with poorly reversible epigenetic features. Accordingly, exhaustion represents a regulated differentiation outcome that balances long‐term immune persistence with controlled effector restraint under conditions of sustained antigen exposure. This framework places exhaustion within the broader landscape of CD8^+^ T cell differentiation, where precursor states generated during physiological immune responses can adopt alternative fates depending on antigen persistence and environmental constraints (Figure 1). Collectively, lineage‐tracing and chromatin studies support a hierarchical differentiation model in which TCF‐1^+^ progenitor cells seed intermediate exhausted populations with preserved effector potential, which subsequently mature into terminally exhausted cells characterised by reduced proliferation and consolidated epigenetic states.

### 1.1 T_PEX_ CD8^+^ T Cells: Identity and Function

T_PEX_ cells occupy the base of the exhaustion hierarchy and form the essential reservoir that sustains the lineage under conditions of chronic stimulation. Their role is not to mediate peak cytotoxicity or cytokine production, but to maintain a renewable progenitor pool that seeds downstream exhausted populations. T_PEX_ cells express TCF‐1 and intermediate levels of PD‐1, lack high levels of terminal differentiation markers such as TIM‐3, and possess a chromatin landscape associated with multipotency and lineage plasticity. They reside predominantly in secondary lymphoid organs, where they receive survival and instructional cues, such as IL‐7, IL‐15, and co‐stimulatory signals, distinct from those acting on intratumoral or tissue‐resident exhausted subsets. Lineage‐tracing and single‐cell trajectory analyses demonstrate that T_PEX_ cells self‐renew and continuously generate differentiated T_EX_ populations during persistent antigen exposure [10, 12, 13, 14].

PD‐1, although inhibitory, does not alone define exhaustion states. Acute effector T cells can transiently upregulate PD‐1 during the peak of activation, and therefore PD‐1 must be interpreted in the context of co‐expressed markers, transcriptional signatures, chromatin accessibility, and anatomical localisation [15]. Co‐expression of TCF‐1, intermediate PD‐1, low TIM‐3, and specific transcriptional modules delineates T_PEX_ cells from both memory precursors and downstream exhausted states [16]. This phenotypic and transcriptional distinction is critical because it underpins the unique functional responsiveness of T_PEX_ cells to immune checkpoint inhibition, enabling cell cycle re‐entry and differentiation into progeny with increased cytokine production and cytotoxic capacity (Table 1).

Cytokine support plays an essential role in maintaining T_PEX_ identity. Common γ chain cytokines, particularly IL‐15, support self‐renewal, metabolic fitness, and survival in chronic viral infection and tumour models [17]. In chronic HBV infection, IL‐15 enhances metabolic balance and preserves progenitor‐like states, improving responsiveness to PD‐1 blockade [18]. These findings highlight the integration of cytokine availability, transcriptional identity, and metabolic programming in defining the T_PEX_ state. Spatial context is also a major determinant of T_PEX_ function. In tumours, lymph node–resident T_PEX_ cells, rather than intratumoral TCF‐1^+^ cells, form the primary reservoir that expands following checkpoint blockade. By contrast, intratumoral cells with progenitor‐like features frequently acquire residency markers and reduced proliferative potential, which limits their contribution to therapeutic responses [19]. This underscores the need to consider anatomical location when interpreting progenitor function.

### 1.2 Transcriptional and Epigenetic Regulation of Exhaustion

Exhausted CD8^+^ T cells exist along a differentiation continuum (Figure 1). In this manuscript, ‘function’ refers specifically to cytokine production (e.g., IFN‐γ, TNF, IL‐2) and cytotoxic capacity (e.g., granule‐mediated killing); when relevant, proliferation is noted separately. T_PEX_ (TCF‐1^+^ PD‐1^+^) maintain proliferative capacity and gives rise to progressively differentiated exhausted populations. These include intermediate T_EX_ cells that retain cytotoxic potential and proliferative responsiveness, and terminal T_EX_ cells characterised by high inhibitory receptor expression, reduced proliferative capacity, and limited epigenetic plasticity [3, 7]. Among exhausted subsets, T_EXint_ cells retain the highest immediate effector potential, whereas T_PEX_ cells prioritise self‑renewal and proliferative capacity, and T_EXterm_ cells display the most restricted cytokine production and proliferation despite preserved antigen recognition. This hierarchical organisation reconciles the coexistence of functional activity with progressive regulatory restraint during chronic stimulation. Exhaustion represents a transcriptionally regulated differentiation programme that reduces effector cytokine production and proliferative potential while preserving antigen recognition and survival under persistent antigen exposure [7]. It is maintained by an integrated regulatory network driven by persistent antigen exposure and cumulative TCR signal integration.

Chronic TCR signalling sustains calcium influx and NFAT activation while AP‐1 activity becomes progressively diminished. This imbalance results in “partnerless NFAT,” which no longer cooperates with AP‐1 to promote effector genes but instead induces TOX and NR4A transcription factors, initiating the exhaustion programme. This shift in NFAT activity forms a central regulatory node controlling exhaustion fate decisions and distinguishes chronic from acute activation. TOX is essential for establishing and maintaining exhaustion [20]. Rather than functioning as a canonical transactivator, TOX modifies chromatin architecture by recruiting epigenetic regulators, histone acetyltransferases, chromatin remodelling complexes, and scaffolding factors to shape exhaustion‐specific enhancer landscapes. These chromatin changes increase accessibility at inhibitory receptor loci, reinforce transcriptional restraint, and limit effector gene expression.

TOX induction becomes self‐sustaining as the programme matures, enabling exhaustion to persist independently of upstream signalling fluctuations [20]. NR4A transcription factors serve as a parallel axis integrating chronic stimulation into exhaustion. NR4A1, NR4A2, and NR4A3 repress effector gene expression by binding AP‐1 motifs and antagonising AP‐1–dependent activation [21, 22]. They also stabilise exhaustion by promoting inhibitory receptor expression and metabolic restraint. Notably, NR4A1 can support T_PEX_ accumulation in tumour settings where NR4A family members are differentially deleted or constrained, revealing context‐dependent roles rather than uniformly exhaustion‐enforcing functions [23].

BATF and IRF4 integrate TCR signal strength, duration, and inflammation into exhaustion. BATF acts as a pioneer factor opening chromatin at exhaustion‐associated regulatory regions, promoting transcriptional programmes that antagonise sustained TCF‑1‐mediated progenitor identity, thereby facilitating the transition from self‑renewing T_PEX_ cells towards more effector‑skewed intermediate exhausted states under continued stimulation [24]. Forced BATF expression can shift differentiation towards effector competent states under chronic antigen exposure [25], demonstrating that interventions targeting this axis may redirect exhausted trajectories. TCF‐1 regulates progenitor identity by maintaining accessible chromatin at memory‐like loci and enabling lineage plasticity [26]. Although TCF‐1 is a canonical mediator of Wnt signalling, its role in exhaustion is largely independent of β‐catenin [27, 28].

Progression from T_PEX_ to T_EXint_ to T_EX term_ is accompanied by increasing chromatin consolidation. Exhaustion‐specific enhancer landscapes persist even after antigen removal in humans recovering from chronic infection [29], producing ‘epigenetic scars’ that restrict functional reprogramming [30]. These findings explain the limited plasticity of T_EX term_ cells and support therapeutic strategies that prioritise preserving, expanding, or redirecting earlier progenitor exhausted populations rather than attempting to reprogramme terminal exhaustion. Tissue specificity further shapes transcriptional circuits. In tumours, NFAT5 responds to hyperosmotic stress and helps drive a TME‐specific exhaustion programme [31]. Such context‐dependent enforcement highlights the importance of integrating environmental cues, such as tissue‐specific metabolic stress and osmotic conditions, into models of exhaustion. Importantly, reduced effector function in exhaustion refers primarily to decreased cytokine production and proliferative capacity relative to effector T cells, whereas antigen recognition and limited cytotoxic activity are preserved.

### 1.3 Metabolic Specialisation and Heterogeneity

Across the exhaustion continuum, downstream T_EX_ populations (particularly T_EXterm_) display diverse metabolic phenotypes that vary across disease contexts and tissue environments, with common features such as mitochondrial dysfunction and reduced spare respiratory capacity emerging under persistent antigen exposure. Persistent antigen exposure imposes metabolic constraints that reinforce exhaustion and shape T_EX_ cell metabolism. Although T_EX_ cells can rely relatively more on oxidative metabolism than rapidly proliferating effector cells, mitochondrial depolarisation and impaired electron transport limit effective respiratory capacity. These metabolic features feed back into transcriptional and epigenetic programmes by limiting metabolite availability for chromatin remodelling enzymes and by promoting sustained expression of exhaustion‐associated genes [32, 33, 34].

Mitochondrial phenotypes vary across disease contexts; for example, virus‐induced T_EX_ cell populations often show increased mitochondrial mass accompanied by functional impairment, whereas tumour‐infiltrating T_EX_ cells frequently exhibit reduced mitochondrial fitness and fragmented organelles [32, 33]. Exhaustion is not metabolically uniform across tissues. During chronic LCMV infection, exhausted cells from spleen, liver, lung, and lymph nodes adopt context‐dependent exhaustion programmes shaped by local oxygen availability, nutrient gradients, exposure to cytokines, and stromal heterogeneity [16]. These findings address concerns that exhaustion was previously portrayed as a single metabolic state; instead, exhaustion is better understood as a shared differentiation programme whose transcriptional core is conserved, but whose functional and metabolic features are shaped by tissue‑specific constraints such as nutrient availability, oxygen tension, and local cytokine signals. They retain greater mitochondrial integrity and metabolic flexibility, attributes that support their proliferative capacity and responsiveness to checkpoint blockade. T_EXint_ cells rely more heavily on glycolysis during proliferation, whereas T_EX term_ cells fail to engage a dominant productive metabolic programme and instead exhibit metabolic rigidity, reduced oxidative capacity, and impaired mitochondrial recovery characterised by a limited ability to restore mitochondrial membrane potential and respiratory function after chronic metabolic stress [32]. IL‐15 enhances T_PEX_ cell maintenance and metabolic health by supporting mitochondrial biogenesis and managing metabolic stress [17]. In chronic HBV infection, IL‐15 promotes progenitor‐like states and improves responsiveness to checkpoint inhibitors [18], consistent with broader observations that IL‐15 supports progenitor exhausted populations under prolonged antigen exposure. These data support interventions aimed at sustaining favourable metabolic conditions that preserve T_PEX_ cell maintenance under chronic antigen exposure.

Metabolic stress in tumours intersects with transcriptional enforcement in unique ways. Hyperosmotic conditions and nutrient restriction activate NFAT5 and further consolidate T_EX_ cell fate [31]. Mitochondrial dysfunction limits the availability of metabolites required for histone demethylation and other chromatin‐modifying processes [29, 32]. These metabolic–epigenetic interactions emphasise why T_EX term_ cells resist re‐differentiation as impaired mitochondrial function and limited metabolite availability constrain chromatin remodelling, thereby reinforcing fixed exhaustion‐associated epigenetic states and necessitating therapeutic focus on earlier differentiation stages. Metabolic adaptations also define tissue‐resident populations. T_RM_ cell differentiation and maintenance depend on controlled metabolic activation [35], with metabolite availability shaping local T cell activation [36]. TGF β‐dependent mechanisms, including those arising from local regulatory populations, can promote the differentiation of T_RM_ cells [37, 38]. These parallels reflect shared reliance on local metabolite availability, cytokine conditioning (including TGF‐β signalling), and restrained activation states that favour tissue adaptation and persistence over maximal effector output in both T_RM_ and T_EX_ cell states.

### 1.4 T_RM_–T_EX_ Cell Convergence and Adaptive Resemblance

Although T_RM_ and T_EX_ cells represent distinct differentiation states with different functional objectives, they display partial convergence driven by shared tissue‐imposed constraints rather than common lineage origin. T_RM_ and T_EX_ populations arise in distinct immunological contexts: T_RM_ cells develop after antigen clearance, whereas T_EX_ cells emerge during persistent antigen exposure. Both can display inhibitory receptor expression, metabolic adaptation, and transcriptional programmes favouring persistence within tissue environments over rapid systemic effector expansion. High‐resolution analyses have shown that intratumoral T_EX_ cells can acquire T_RM_‐associated surface features such as CD69 and CD103 as part of local tissue adaptation, alongside elevated inhibitory receptors, including PD‐1 and CTLA‐4, as well as modified adhesion properties and suppressed expression of egress receptors [19] (Table 1). These similarities reflect convergent responses to environmental stress rather than shared developmental origin. T_RM_ and T_EX_ cells both respond to TGF β, which restricts migration, influences transcriptional restraint, and promotes local adaptation.

Metabolic parallels also emerge; tissue‐resident cells rely on local metabolite availability [36] and maintain controlled activation states in response to epithelial signals [35]. Local regulatory populations and cytokine environments shape residency, with TGF β‐dependent programmes promoting establishment of T_RM_ subsets [37, 38]. These parallels emphasise environmental conditioning as a shared determinant of differentiation. However, the two states diverge significantly. T_RM_ cells are protective sentinels positioned for rapid pathogen recall, whereas T_EX_ populations engage in sustained antigen surveillance with reduced cytokine production and cytotoxic capacity relative to effector cells. Their epigenetic landscapes differ, with T_RM_ preserving memory‐like chromatin poised for recall and T_EX_ establishing exhaustion‐specific accessibility patterns that limit plasticity [29]. In tumour immunity, T_RM_‐like T_EX_ cell subsets persist and can limit local tumour growth but respond poorly to checkpoint blockade due to residency‐imposed constraints. In contrast, lymph node T_PEX_ cells deliver the proliferative burst required for effective therapy [19]. These observations reinforce the importance of anatomical and environmental context in interpreting T cell identity and function.

### 1.5 Therapeutic Implications

Understanding exhaustion as a structured differentiation programme reframes therapeutic strategies for chronic infection and cancer by shifting emphasis towards preserving and leveraging progenitor exhausted states rather than attempting to reverse terminal exhaustion. Immune checkpoint blockade predominantly expands T_PEX_ cells, which subsequently generate proliferative T_EXint_ progeny with increased effector potential [6, 12], which retain proliferative capacity and epigenetic plasticity. This expansion of progenitor populations subsequently generates waves of proliferative T_EXint_ cells with increased effector potential, whereas terminal T_EX_ cells exhibit limited functional reinvigoration [6]. Anatomical localisation influences therapeutic outcomes. In tumour settings, lymph node T_PEX_ cells, not intratumoral progenitor‐like cells, mediate responses to PD‐1 blockade [19]. This dependence on lymph node progenitors suggests that therapies delivering cytokines, co‐stimulatory signals, or checkpoint inhibitors to tumour‐draining lymph nodes could enhance efficacy. Consistent human data further link progenitor‐like states with favourable clinical responses to immune checkpoint blockade [39].

Cytokines can be used as tools to steer T_PEX_ differentiation. IL‐2 can synergise with PD‐1 blockade to promote the development of high‐quality effector cells [39]. IL‐15 expands T_PEX_ cells and enhances their metabolic resilience [17]. New strategies aim to engineer cytokines with selective targeting of progenitor subsets to avoid toxicity and improve durability. Adoptive cell therapies, including CAR T cells, increasingly focus on engineering T_PEX_‐like profiles. Limiting ex vivo stimulation preserves TCF‐1 expression and progenitor‐like features [40], while metabolic modulation, CRISPR‐mediated deletion of exhaustion‐enforcing transcription factors [21], and incorporation of cytokine circuits aim to maintain stem‐like potential during expansion. These designs shift the focus from generating terminal effector cells to producing durable, self‐renewing T cell populations capable of sustained anti‐tumour activity. A major consideration in therapeutic design is the risk associated with manipulating the exhaustion programme. Excessive T_PEX_ expansion could exacerbate autoimmunity and tissue damage by overwhelming the regulatory restraint that exhaustion evolved to impose under conditions of sustained antigen exposure. The context‐specific roles of transcriptional regulators, such as NFAT5 in tumours [31], highlight the importance of tailoring treatments to particular microenvironments.

### 1.6 Conclusions and Outstanding Questions

Exhaustion represents an orchestrated differentiation programme that enables sustained control of persistent antigen while preventing immunopathology. T_EX_ subsets balance cytotoxic activity with restraint, and T_PEX_ cells serve as regenerative hubs capable of responding to immunotherapy through proliferative expansion and generation of downstream exhausted progeny. The partial convergence between T_RM_ and T_EX_ cells reflects shared adaptation to restrictive environments rather than shared lineage. Several key questions remain.

While T_RM_ and T_EX_ cells share adaptive features driven by tissue residency and environmental constraint, they represent distinct differentiation outcomes with differing functional objectives and degrees of plasticity. Conceptually related strategies are also evident in other T cell lineages exposed to prolonged antigenic stimulation, such as T follicular helper (Tfh) cells, which maintain antigen sensitivity and regulated effector output within specialised lymphoid niches. Current understanding of the relative functional overlap and divergence between these states remains limited, in part due to differences in experimental systems, anatomical localisation, and temporal resolution. Future studies combining lineage tracing, spatial multi‐omics, and functional perturbation across tissues will be required to define how persistent antigen exposure shapes convergent or divergent adaptive T cell trajectories.

It is unclear how stable the T_PEX_ compartment is under physiological cytokine and stromal conditions in humans. The factors that determine whether progenitors remain lymph node restricted or infiltrate peripheral tissues are incompletely defined. Tissue‐specific enforcement mechanisms, such as NFAT5 in tumours, raise the possibility of microenvironment‐restricted therapeutic modulation. The extent to which metabolic and epigenetic coupling can be modulated to enhance cytokine production and cytotoxic capacity without compromising protective restraint is unknown.

Integration of lineage‐tracing, epigenetic profiling, and spatial single‐cell analyses now defines exhaustion not as a static phenotype but as a dynamic differentiation system shaped by antigen persistence, tissue microenvironment, and metabolic constraint. The role of T_RM_‐like T_EX_ cells in sustaining long‐term tumour control outside the context of checkpoint therapy also remains to be defined. Addressing these questions will refine strategies that enhance progenitor fitness, preserve diversity within the exhaustion continuum, and leverage the adaptive logic of exhaustion for improved clinical outcomes. Ultimately, these insights suggest that effective immunotherapies will depend less on reversing terminal exhaustion and more on preserving, expanding, or engineering progenitor exhausted populations capable of sustaining durable immune responses.

## Author Contributions

The authors contributed equally to the conception and writing process.

## Conflicts of Interest

C.F. and M.V. are co‐inventors on a patent application related to T_RM_‐based tumour therapy.

## Data Availability

This is a review, and no additional data were acquired.
